# The effect of host social system on parasite population genetic structure: comparative population genetics of two ectoparasitic mites and their bat hosts

**DOI:** 10.1186/1471-2148-14-18

**Published:** 2014-01-30

**Authors:** Jaap van Schaik, Gerald Kerth, Nadia Bruyndonckx, Philippe Christe

**Affiliations:** 1 Department of Behavioural Ecology and Evolutionary Genetics, Max Planck Institute for Ornithology, Eberhard-Gwinner-Strasse, 82319 Seewiesen, Germany; 2 Zoological Institute & Museum, Greifswald University, J.-S.-Bach-Str. 11 / 12, D-17489 Greifswald, Germany; 3 Department of Ecology and Evolution, University of Lausanne, Biophore, CH-1015 Lausanne, Switzerland

**Keywords:** Coevolution, Host-parasite interaction, Local adaptation, Social system, *Myotis myotis*, *Spinturnix myoti*, *Myotis bechsteinii*, *Spinturnix bechsteini*

## Abstract

**Background:**

The population genetic structure of a parasite, and consequently its ability to adapt to a given host, is strongly linked to its own life history as well as the life history of its host. While the effects of parasite life history on their population genetic structure have received some attention, the effect of host social system has remained largely unstudied. In this study, we investigated the population genetic structure of two closely related parasitic mite species (*Spinturnix myoti* and *Spinturnix bechsteini*) with very similar life histories. Their respective hosts, the greater mouse-eared bat (*Myotis myotis*) and the Bechstein’s bat (*Myotis bechsteinii*) have social systems that differ in several substantial features, such as group size, mating system and dispersal patterns.

**Results:**

We found that the two mite species have strongly differing population genetic structures. In *S. myoti* we found high levels of genetic diversity and very little pairwise differentiation, whereas in *S. bechsteini* we observed much less diversity, strongly differentiated populations and strong temporal turnover. These differences are likely to be the result of the differences in genetic drift and dispersal opportunities afforded to the two parasites by the different social systems of their hosts.

**Conclusions:**

Our results suggest that host social system can strongly influence parasite population structure. As a result, the evolutionary potential of these two parasites with very similar life histories also differs, thereby affecting the risk and evolutionary pressure exerted by each parasite on its host.

## Background

In the evolutionary arms race between hosts and their parasites, the interacting species may use immunological, physiological and behavioural adaptations [1,2]. Hosts commonly use behavioural adaptations to avoid exposure, or actively remove, parasites [3]. In addition behavioural adaptations may also affect parasite population structure, thereby potentially reducing parasite intensity [1], and ultimately their evolutionary potential, classically defined as the ability to incorporate genotypes able to outcompete those put forward by the opponent [4]. On the population level, a parasite’s genetic structure is closely linked to the life histories of both interacting species [5], as well as to the social system of the host [6]. Specifically, the relative rate of dispersal, and thereby gene flow [6], as well as the relative strength of genetic drift [7,8], are important in determining host-parasite coadaptation dynamics. Therefore, comparisons of population genetic structure across multiple host-parasite pairs where hosts differ in key life-history and/or social system traits, will help elucidate how these factors affect microevolutionary host-parasite dynamics.

Relative dispersal rates of hosts and parasites have been studied in a number of systems. For instance, in the black-legged kittiwake and a parasitic tick, parasite populations were found to be much more spatially structured than those of their hosts [9]. This difference was attributed to the fact that parasite dispersal was limited to the breeding season of the hosts, and therefore dispersal of the host outside of this period did not result in concurrent parasite dispersal [9]. In contrast, in a comparison of dispersal rates between two shearwater species and three parasitic lice, parasite gene flow was found to be much higher than that of its host, which was attributed to the transmission of parasites at communal wintering grounds where no host gene flow took place [10]. These examples highlight the fact that relative dispersal is dependent on the intricate interaction between the life histories of both species. In cases where gene flow in host and parasite are comparable, genetic drift can also substantially affect host-parasite coevolutionary dynamics as it can impede adaptation; even in cases where standing genetic variation is abundant in both species [8]. This is especially relevant for parasites of vertebrate hosts, where meta-population dynamics often lead to patchily distributed parasite populations. As a result parasite populations often experience extinction events and strong population bottlenecks eg. [11]. Unfortunately, comparative studies investigating the role of host social system in shaping parasite population genetic structure are rare.

In this study, we investigate the population genetic structure of two ectoparasitic mite species that parasitize two closely related bat hosts with differing social systems*.* The mite species (*Spinturnix myoti* and *Spinturnix bechsteini*) are closely related and have indistinguishable life histories [12,13]. As a result, we expect any differences observed in their microgeographic population genetic structure to be primarily the result of differences in the social system of their hosts, the greater mouse-eared bat (*Myotis myotis*) and the Bechstein’s bat (*Myotis bechsteinii*).

The two host species share similar life-history traits but show substantial differences in their social systems, which include their social organization, social structure and mating system (Table 1). Both are long-lived, monotocous, non-migratory European vespertilionid bat species that follow the temperate cycle. In summer, they form exclusively female maternity colonies, which however differ between the two species in size, location, stability and degree of philopatry (Table 1). Females from different maternity colonies of *M. myotis* sometimes visit other maternity colonies and occasionally co-localize with maternity colonies of closely related *Myotis blythii,* whereas females from *M. bechsteinii* maternity colonies do not interact with one another or with other species. Males of *M. myotis* and *M. bechsteinii* disperse from their natal colony and are solitary throughout the summer [14,15]. In the autumn mating season, male *M. myotis* form temporary harems in August and September where several females roost in direct contact with a male for one or more days. In contrast, *M. bechsteinii* mate at swarming sites where the sexes meet very briefly during the night [16]. In winter both species hibernate at underground sites, but again differ in degree of aggregation and body contact, where *M. myotis* may form large clusters whereas *M. bechsteinii* roosts solitarily [17]. Finally, the species differ in the distance travelled between summer and winter roosts. *M. myotis* is considered a regional migrant, easily travelling over 50 km, whereas *M. bechsteinii* is considered sedentary, generally not travelling over 30 km [18]. Both species have recolonized the current study area since the last glacial maximum. However, for *M. myotis* there is the possibility of admixture as multiple glacial refugia have been identified (Iberia and Italy; [19]), while all *M. bechsteinii* in Central Europe are believed to originate from the Balkan region [20]. Both species are parasitized by ectoparasitic mites of the genus *Spinturnix*, which typically show strong cospeciation with their bat hosts [21].

**Table 1:** Key differences in social system of the two host species

	**Greater mouse-eared bat**	**Bechstein’s bat**	**Predicted effect on parasite population genetic structure**	**Reference**
	***Myotis myotis***	***Myotis bechsteinii***		
**Social organization**				
Colony size	Large (50–2000)	Small (10–50)	Larger colonies lead to less genetic drift	[17]
Female natal philopatry	High; but occasional exchange of individuals between colonies	Very high; almost no exchange of individuals between colonies	Lower philopatry leads to more parasite transmission	[31,53]
Roost fidelity	High; one site (building/cave) throughout summer	Very low; frequent roost switching (tree cavities) and fission-fusion dynamics	Fission-fusion dynamics may increase genetic drift because colonies split into subgroups	[17,54]
Hibernation	Free-hanging; solitary or clustered	In crevices (mostly solitary)	Solitary roosting reduces parasite transmission	[17]
**Mating system**	Temporary harems (extensive contact) and/or swarming (little contact)	Swarming (little contact)	Temporary harems increase parasite transmission	[14,16]

Overview of the key differences in social system between the greater mouse-eared bat (*Myotis myotis*) and Bechstein’s bat (*Myotis bechsteinii*), and the expected effects thereof on the population genetic structure of their parasites.

*Spinturnix* wing mites live exclusively on membranes of bats, reproduce sexually, are haematophagous and cannot survive off of their host for more than a few hours [22]. Therefore, while dispersal of *Spinturnix* mites is extensive within bat maternity colonies, where female bats live in close body contact, little (*M. myotis*) or no (*M. bechsteinii*) transmission can occur between different maternity colonies in summer [23]. It is expected that horizontal transmission of mites between colonies occurs during bat mating and hibernation periods. Bat maternity colonies constitute optimal conditions for parasite reproduction, and parasites show a strong preference for female and juvenile hosts [24]. Mite abundance subsequently strongly decreases in autumn and throughout hibernation, and it is supposed that they are not able to reproduce during this time [25]. Mites are believed to impose a substantial cost on their hosts during the maternity period [26]. For example, in *M. myotis* individuals experienced increased oxygen consumption and weight loss when experimentally infected [27]. Mite prevalence and intensity is much higher in *M. myotis* (intensity per female 12–20; [28]) compared to *M. bechsteinii* (intensity per female 1–10; [29]). *S. bechsteini* is strictly host specific. *S. myoti* is found on *M. myotis* as well as its sister species *M. blythii* (which also has an identical social system; [17]). In choice experiments *S. myoti* shows a clear preference for *M. myotis* and it is presumed that *M. myotis* is the main host species [30]. A previous study of mtDNA sequence data in *S. bechsteini* found strong spatial differentiation between bat colonies and a high temporal turnover in the haplotypes found per bat colony, suggesting frequent local extinction and recolonisation [23].

By comparing the genetic diversity, gene flow and genetic drift of the selected parasite species, we aim to assess the consequences that the differing social systems of the hosts have on the evolutionary potential of the parasites. In accordance with Nadler [5], we predict that the larger colony size and increased contact rates among colonies of *M. myotis* will result in a less genetically structured population in *S. myoti*, and allow for substantial gene flow between colonies. In contrast, we expect the mite population of *M. bechsteinii* to be highly sub-structured due to reduced dispersal opportunities and strong genetic drift as a result of the smaller and demographically more isolated host colonies.

## Methods

### Previously analysed samples

To compare host and parasite population genetic structure, we have combined newly generated datasets for both parasites (*S. myoti*: mtDNA *cytb* sequence data and 8 nucDNA microsatellites; *S. bechsteini*: 5 nucDNA microsatellites), with previously generated datasets for one of the mite species (*S. bechsteini*: mtDNA *cytb*) and both hosts (mtDNA and nucDNA), supplemented for *M. myotis* with two additional colonies for this study. An overview of the markers and samples previously analysed is given below as well as in the supplemental materials (Additional file 1).

For *M. myotis*, four summer maternity colonies had been previously genotyped for ten microsatellites and sequenced for the second hypervariable domain (*HVII*) of the mtDNA control region [31]. For *M. bechsteinii*, individuals from ten maternity colonies had been genotyped for eight nucDNA microsatellites as well as two mitochondrial microsatellites [20,32]. For *S. bechsteini*, mites from thirteen bat maternity colonies had been previously sequenced for a 513 bp fragment of Cytochrome B (*cytb*; mtDNA) [23].

### Sample collection and DNA extraction

**Figure 1:**
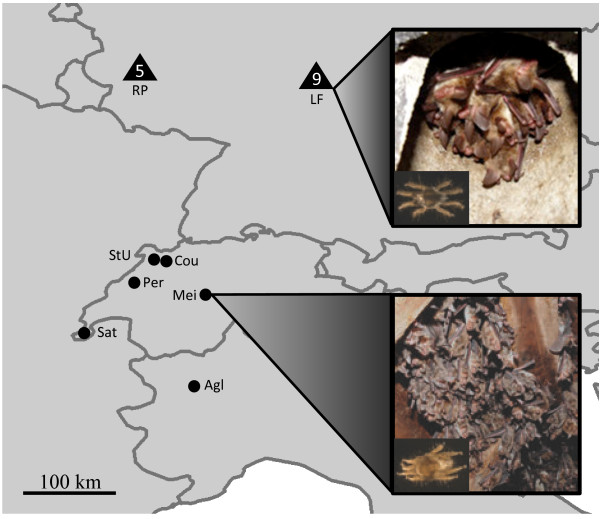
**Sampling map.** Map of Central Europe showing the sampling locations for *Myotis myotis*/*Spinturnix myoti* (circles), and sampling regions for *Myotis bechsteinii*/*Spinturnix bechsteini* (triangles). For the latter pairing*,* the number of sampled colonies within a region is indicated within the triangle because all colonies within a region are located in close proximity to one another. All colony and region names correspond to the abbreviations given in Table 2. For each species, a picture of the typical roosting association is shown with an inset of the studied mite species (picture credits *M. bechsteinii*: GK, *M. myotis*: PC, mites: GK & JvS).

We sampled *S. myoti* in six *M. myotis* maternity colonies in Switzerland and Northern Italy in August 2004 and 2005 (Figure 1, Table 2a). Colony size estimates were made inside the roost and bats were caught either directly inside the colonies’ roosts during the day, or upon emergence from the roost entrance at night. Geographic distances between colonies ranged from 16 to about 200 km, which is comparable to the range *M. myotis* is known to disperse between summer and winter roosts [18]. Twenty mites per bat colony, each originating from a different bat, were used for the genetic analyses.

**Table 2:** Descriptive statistics for all species

**a) *S. myoti***						**Mite mtDNA**			**Mite microsatellites**				
**Colony name**		**Year**	**Lat**	**Long**	**n**	**N**	**π**	**N**_**A**_	**K**	**H**_**o**_	**H**_**e**_	**P**_**all**_	**F**_***is***_
Agliè (Agl)		2005	45.3671	7.767	20	14	0.708	17.875	15.955	0.61	0.868	22	0.264
Courtételle (Cou)		2004	47.341	7.3178	19	13	0.701	18.125	16.752	0.755	0.904	11	0.191
Meiringen (Mei)		2005	46.7296	8.1812	18	7	0.777	13.125	12.743	0.546	0.871	6	0.209
Perreux (Per)		2004	46.9479	6.8179	20	10	0.874	18	16.121	0.733	0.898	13	0.32
Satigny (Sat)		2005	46.2143	6.0357	20	8	0.536	15.5	14.122	0.663	0.872	16	0.398
St-Ursanne (StU)		2005	47.3647	7.1537	20	14	0.759	17.5	15.787	0.695	0.89	11	0.244
**b) *M. myotis***			**Host mtDNA**					**Host microsatellites**					
**Colony name**		**Colony size**	**n**	**N**	**π**	**n**		**N**_**A**_	**K**	**H**_**o**_	**H**_**e**_	**P**_**all**_	**F**_***is***_
Agliè (Agl)		400	20	4	1.817	20		8.2	8.71	0.735	0.723	5	-0.017
Courtételle (Cou)		800	20	2	0.033	20		9	8.39	0.785	0.761	2	-0.031
Meiringen (Mei)		80	20	2	0.062	20		9	7.43	0.795	0.772	4	-0.029
Perreux (Per)		200	20	3	0.161	20		9.9	8.16	0.79	0.789	9	-0.002
Satigny (Sat)		190	18	3	1.993	20		9.3	7	0.789	0.763	3	-0.034
St-Ursanne (StU)		200	20	1	0	20		9.7	9.03	0.733	0.763	6	0.039
**c) *S. bechsteini***						**Mite mtDNA**			**Mite microsatellites**				
**Region**	**Colony name**	**Year**	**Lat**	**Long**	**n**	**N**	**π**	**N**_**A**_	**K**	**H**_**o**_	**H**_**e**_	**P**_**all**_	**F**_***is***_
Lower Frankonia (LF)	Blutsee (BS)	2007	49.4331	9.4958	41	3	0.18	5.2	3.981	0.736	0.645	1	-0.13
	Einsiedeln (ES)	2002	49.5407	9.5641	16	2	0.32	6.8	5.806	0.727	0.703	2	0
		2007			11	4	0.67	6.8	6.237	0.739	0.754	0	0.069
	Guttenberg 2 (GB2)	2002	49.4443	9.5154	15	3	0.6	12	9.354	0.747	0.864	10	0.169
		2007			20	2	0.27	6.6	5.24	0.747	0.706	2	-0.032
	Gramschatz 1(GS1)	2002	49.5409	9.5842	25	3	0.46	8.2	6.133	0.677	0.75	2	0.118
		2007			22	6	0.77	10.2	7.822	0.757	0.843	2	0.125
	Höchberg (HB)	2002	49.4705	9.5201	21	5	0.49	7.4	5.9	0.632	0.743	3	0.175
		2007			20	1	0	5.2	4.375	0.627	0.631	0	0.033
	Irtenberg 3 (IB3)	2002	49.4308	9.5016	23	1	0	5	4.453	0.753	0.677	1	-0.089
		2007			38	1	0	4.6	3.598	0.579	0.584	0	0.023
	Reutholz (RT)	2007	49.737	9.861	22	3	0.66	9.8	7.601	0.696	0.809	2	0.164
	Steinbach (SB)	2007	49.4215	9.4442	21	1	0	4.2	3.833	0.657	0.572	1	-0.126
Rhineland-Palatine (RP)	Altrich 4 (AL4)	2007	49.9636	6.8726	14	2	0.14	5.4	4.728	0.643	0.662	2	0.065
	Bitburg (BI)	2007	49.9726	6.4793	22	3	0.44	7.4	5.735	0.609	0.753	1	0.216
	Duppach (DU)	2007	50.2685	6.5507	24	6	0.72	8.2	6.549	0.67	0.772	2	0.154
	Longuich (LO)	2007	49.7916	6.7514	25	3	0.67	6.6	5.01	0.701	0.71	1	0.033
	Orenhofen (OH)	2007	49.9115	6.6781	22	1	0	2.6	2.255	0.345	0.352	0	0.043
**d) *M. bechsteinii***			**Host mt msats**						**Host microsatellites**				
**Colony name**		**Colony size**	**n**	**N**	**π**	**n**		**N**_**A**_	**K**	**H**_**o**_	**H**_**e**_	**P**_**all**_	**F**_***is***_
Lower Frankonia (LF)	Blutsee (BS)	15-19	30	3.5	2.22	30		11.1	5.07	0.783	0.802	1	0.057
	Einsiedeln (ES)	20-24	10	1	1	10		7.1	4.63	0.85	0.769	0	-0.052
	Guttenberg 2 (GB2)	35-39	70	2	1.55	70		12.6	4.95	0.801	0.825	2	0.041
	Gramschatz 1 (GS1)	40-44	93	1.5	1.08	93		13	4.83	0.828	0.81	4	-0.014
	Höchberg (HB)	20-24	57	4.5	2.45	57		11.6	4.86	0.806	0.799	1	0.008
	Irtenberg 3 (IB3)	25-29	16	1.5	1.35	16		9.5	4.99	0.844	0.799	2	-0.024
	Reutholz (RT)	40-44	17	1	1	17		9.3	4.95	0.79	0.794	2	0.029
	Steinbach (SB)	20-24	34	1	1	34		9.3	4.57	0.825	0.792	1	-0.007
Rhineland-Palatine (RP)	Bitburg (BI)	40-44	9	1	1	9		6.8	4.85	0.819	0.795	3	0.028
	Duppach (DU)	45-49	20	1.5		1.3		9.1	4.86	0.856	0.804	1	-0.039

Description of molecular variability in a) *Spinturnix myoti*, b) *Myotis myotis* c) *Spinturnix bechsteini* d) *Myotis bechsteinii*. The following parameters are recorded: latitude (Lat), longitude (Long), estimated number of bats in the colony (colony size), number of individuals sequenced/genotyped (n), total number of haplotypes (N), nucleotide diversity (π), the mean number of microsatellite alleles per locus (N_A_), allelic richness (K), observed (H_o_) and expected (H_e_) heterozygosities, the number of private alleles per colony (P_all_), and the inbreeding coefficient (F_is_). For *S. myoti* and *S. bechsteini* the number of individuals sequenced and genotyped was the same for each colony.

*S. bechsteini* samples were collected from 13 *M. bechsteinii* maternity colonies in 2002 and in 2007 in two spatially distant regions (±200 km) in Germany, Lower Franconia (LF) and Rhineland-Palatinate (RP; Figure 1). As *M. bechsteinii* has not been recorded to disperse over 73 km [18] direct dispersal of either host or parasite between regions is not expected. At the same time, within regions, inter-colony distances are again comparable to the expected dispersal distance between summer and winter roosts. We divided the available 18 sampling events from 13 colonies into three main subgroups based on spatial and temporal characteristics (LF in 2002: 100 mites from 5 bat colonies; LF in 2007: 195 mites from 8 bat colonies; RP in 2007: 107 mites from 5 bat colonies). The number of sampling events in each subgroup is less than were analysed for mtDNA [23] as all events with less than 10 samples were discarded in order to ensure sufficient sample sizes for all nucDNA analyses. Samples from 2002 were only used for the temporal analysis, whereas all other comparisons were performed using only the samples from 2007. In all cases, all bats within a (bat-box) roost were caught and sampled for mites, and colony size estimates reflect the number of bats present in the colony. All mites collected from a colony were used for the genetic analysis.

For both mite species, individuals were removed from the bat’s wing and tail membrane using soft forceps and stored in 90% ethanol prior to DNA extraction. Collected mite specimens were individually rehydrated for two hours in 200 μl of sterile water before being crushed in liquid nitrogen. Total DNA was isolated from each individual mite using a standard proteinase K-phenol chloroform method [33].

### Additional host samples

For the two newly analysed *M. myotis* maternity colonies (Satigny, St-Ursanne), wing tissue punches from 20 bats were obtained with a sterile biopsy punch of the wing membrane Ø 2 mm [34]. For these colonies samples were taken concurrently with mite samples, for the other bat maternity colonies samples were collected and analysed previously [31]. Samples were preserved, extracted, and sequenced for the second hypervariable domain (*HVII*) of the mtDNA control region and 10 microsatellites (A13, B11, B22, C113, E24, F19, G9, H19, H29 and G30) as described in Castella et al. [31].

### Amplification

Amplification of *Cytochrome b* (*cytb*) for *S. myoti* was performed according to the protocol described in Bruyndonckx *et al.*[21] using the primer pair C1-J-2183 and C1-J-2797mod [35]. Microsatellite loci for both species were developed from an enriched genomic library of *S. myoti*. For *S. myoti*, eight microsatellite loci were established (SM7, SM11, SM13, SM17, SM18, SM19, SM51 and SM55), where for *S. bechsteini* only five microsatellites could be successfully amplified (SM11, SM16, SM17, SM18 and SM35) [GenBank: JF288840- JF288850] [36]. Amplification protocol and multiplex configuration were as developed in van Schaik *et al*. [36].

### Mite mtDNA genetic analysis

Haplotype diversity (H) and nucleotide diversity (*π*) were calculated using Arlequin 3.5 [37]. A statistical parsimony network was computed using TCS 1.21 [38]. An analysis of molecular variance, performed in Arlequin 3.5, was used to examine the genetic structure and estimate pairwise Φ-statistics among all populations. To test whether Φ-statistics were sensitive to distances between haplotypes, we performed the same analyses based only on haplotypic frequencies. Isolation by distance in mite populations was tested through the correlation between matrices of Φ_ST_/(1- Φ_ST_) values and log transformed geographic distance using Mantel tests (10,000 permutations) in FSTAT 2.9.3 [39]. Geographic distances were calculated as the shortest linear distance connecting the populations.

### Mite microsatellite genetic analysis

For both mite species we checked for the presence of null alleles, allelic dropout, and stuttering using MicroChecker 2.2.5 [40]. We also checked for deviations from Hardy-Weinberg equilibrium and linkage disequilibrium using Genepop on the web 4.0.10 [41]. We calculated number of alleles per locus, allelic richness, observed and expected heterozygosity, F_IS_, and pairwise F_ST_-values using Fstat 2.9.3 [39]. Number of private alleles was calculated using Genalex 6 [42]. To assess the level of genetic differentiation of mites within and between bat colonies, single-level and hierarchical AMOVAs were performed in Arlequin 3.5 [43]. Isolation by distance was measured by comparing matrices of F_ST_ / (1- F_ST_) and log transformed geographic distance using Mantel tests (10,000 permutations) in FSTAT 2.9.3 [39]. Sequential Bonferroni corrections were used to compute the critical significance levels for simultaneous statistical tests. Pairwise F’_ST_ and G”_ST_-values were calculated using Genodive 2.0b23 [44].

To investigate temporal differentiation of mite populations within a colony, mites from five colonies of *M. bechsteinii* were sampled twice (2002 and 2007) and compared using pairwise F_ST_-values and a Mantel test in FSTAT 2.9.3 [39].

### Parasite and host comparison

Summary statistics, diversity indices and population differentiation were calculated for both hosts from existing datasets and the two newly analysed *M. myotis* colonies as described above for mites. To compare parasite and host genetic distances we used a partial Mantel test (10000 permutations) correcting for geographic distance. For comparison of parasite and host mitochondrial genetic distance, we used Φ_ST_ / (1- Φ_ST_), and nuclear genetic distance was compared using F_ST_ / (1 - F_ST_) [45]. A structure analysis was performed in STRUCTURE 2.3.3 [46] for all species using only the nucDNA microsatellites, applying an admixture model without location as a prior. For *S. myoti* and *M. myotis*, we selected a range of K from 1 to 10, and for *S. bechsteini* and *M. bechsteinii* from 1 to 15. Five iterations per K were run with a burn-in and run length of 200 000 and 1 000 000 repetitions respectively. The most probable K was inferred using the ΔK method of Evanno et al. [47]. Finally, one-tailed Spearman’s rank correlation tests were performed using R[48] to test for a positive correlation between mite diversity indices (H, π, K, H_O_, H_E_) and host colony size.

## Results

### *M. myotis* genetic data

The additional host mitochondrial analysis revealed that all *HV2* haplotypes corresponded to previously published haplotypes [19,31,49] (Table 2b). As expected, *M. myotis* bat colonies showed high mtDNA differentiation (Φ_ST_ = 0.56, p < 0.001) and, despite being statistically significant, low nucDNA (F_ST_ = 0.015, p < 0.001) differentiation, indicating strong female philopatry and male-biased dispersal. Descriptive statistics for all colonies can be found in Table 2b; results are similar to those previously published by Castella *et al.*[31].

### *S. myoti* mtDNA

Among the 117 *S. myoti* mites sequenced, 49 different *cytb* haplotypes were detected. The 697 aligned nucleotides consisted of 49 variables sites, of which 18 were parsimony-informative. All haplotypes were deposited in GenBank under accession numbers [KJ174107-KJ174155].

**Figure 2:**
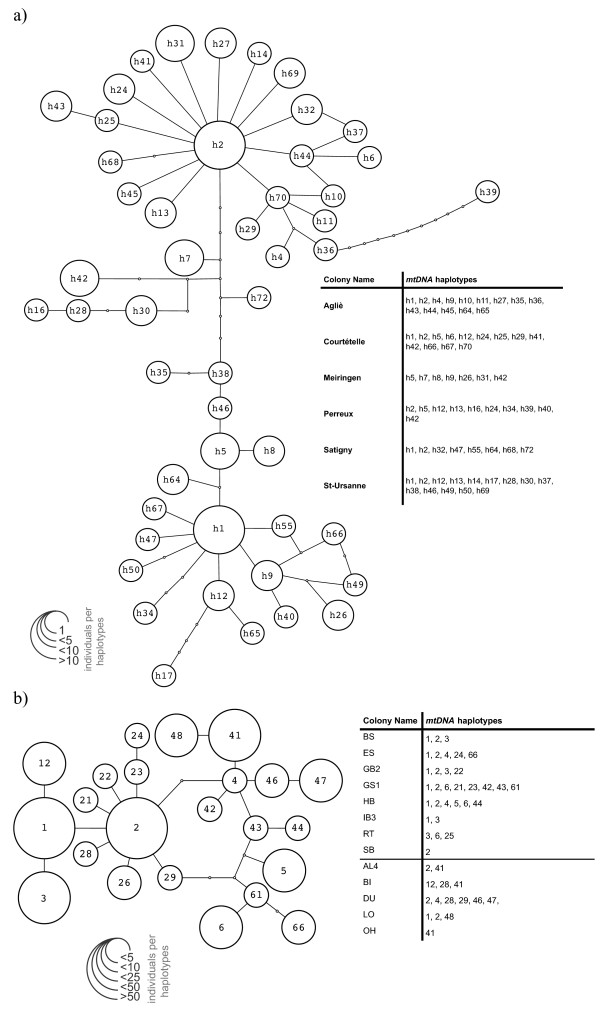
**Haplotype network for both parasite species.** Haplotype network for **(a)** the 49 haplotypes determined by sequencing the *cytochrome b* (*cytb*) of 119 *Spinturnix myoti*, and **(b)** the 23 haplotypes for *cytochrome b* (*cytb*) for the 402 *Spinturnix bechsteini* included in this comparison, as previously analysed in Bruyndonkx *et al.* (2009b). The size of circles is proportional to the number of individuals sharing the same haplotype. For each species, the list of which haplotypes were found in each colony is given as an inset.

The haplotype network of *S. myoti* presents two star-like patterns with some haplotypes in between (Figure 2a). Two haplotypes were the most represented: h1 was present in 12% of individuals and h2 in 16.2%. Nine haplotypes were shared between bat colonies and 40 were private haplotypes. The number of haplotypes per population ranged from 7 to 14 (mean = 11, Table 2a), and nucleotide diversity from 0.536 to 0.874 (mean = 0.726; Table 2a). *S. myoti* populations showed low pairwise differentiation (mean Φ_ST_ = 0.012; Table 3a), and only Satigny was significantly differentiated from two other populations (Meiringen, Agliè). A mantel test revealed no significant correlation between genetic and geographic distances (r^2^ = 0.02, p = 0.64).

**Table 3:** **Pariwise Φ**_**ST **_**and F**_**ST**_**-values for all species**

**a) *S. myoti***														
**Mite Φ**_**ST**_**\F**_**st**_			**Agliè**		**Courtételle**		**Meiringen**		**Perreux**		**Satigny**		**St-Ursanne**	
Agliè			-		0.014*		0.018*		0.014*		0.026*		0.016*	
Courtételle			−0.02		-		0.004		0.013		0.004		0.002	
Meiringen			0.05		0.02		-		0.01		0.015*		0.004	
Perreux			−0.01		−0.04		0.01		-		0.015		0.009	
Satigny			0.11*		0.04		0.08*		0.05		-		0.012*	
St-Ursanne			0.03		−0.01		0.03		−0.01		0.02		-	
**b) *M. myotis***														
**Host Φ**_**ST**_**\F**_**ST**_			**Agliè**		**Courtételle**		**Meiringen**		**Perreux**		**Satigny**		**St-Ursanne**	
Agliè			-		0.043*		0.057*		0.034*		0.035*		0.036*	
Courtételle			0.72*		-		*0.016*		*−0.001*		*0.009*		*−0.003*	
Meiringen			0.71*		0.04		-		*0.0004*		*0.007*		*0.014*	
Perreux			0.70*		0.15*		0.12*		-		*0.006*		*0.003*	
Satigny			0.37*		0.21*		0.20*		0.18*		-		*0.002*	
St-Ursanne			0.73*		0		0.05		0.18*		0.22*		-	
**c) *S. bechsteini***														
		**LF**									**RP**			
	**Mite Φ**_**ST**_**\F**_**st**_	**BS**	**ES**	**GB2**	**GS1**	**HB**	**IB3**	**RT**	**SB**	**AL4**	**BI**	**DU**	**LO**	**OH**
LF	Blutsee	-	0.217*	0.255*	0.202*	0.316*	0.126*	0.153*	0.351*	0.260*	0.243*	0.185*	0.275*	0.406*
	Einsiedeln	0.54*	-	0.174*	0.084*	0.184*	0.275*	0.068*	0.221*	0.130*	0.123*	0.145*	0.177*	0.365*
	Guttenberg 2	0.73*	0.51*	-	0.131*	0.283*	0.307*	0.116*	0.315*	0.180*	0.186*	0.186*	0.215*	0.421*
	Gramschatz 1	0.22*	0.16*	0.36*	-	0.159*	0.226*	0.052*	0.213*	0.133*	0.130*	0.079*	0.098*	0.312*
	Höchberg	0.88*	0.75*	0.87*	0.60*	-	0.348*	0.171*	0.299*	0.228*	0.210*	0.204*	0.248*	0.422*
	Irtenberg 3	0.90*	0.83*	0.91*	0.69*	1.00*	-	0.158*	0.372*	0.322*	0.284*	0.213*	0.301*	0.458*
	Reutholz	0.62*	0.33*	0.53*	0.27*	0.66*	0.53*	-	0.233*	0.121*	0.149*	0.113*	0.135*	0.309*
	Steinbach	0.87*	0.72*	0.110	0.55*	1.00*	1.00*	0.66*	-	0.250*	0.244*	0.267*	0.239*	0.475*
RP	Altrich 4	0.83*	0.61*	0.77*	0.50*	0.94*	0.96*	0.56*	0.94*	-	0.214*	0.173*	0.163*	0.314*
	Bitburg	0.72*	0.47*	0.64*	0.40*	0.77*	0.83*	0.45*	0.78*	0.60*	-	0.138*	0.181*	0.379*
	Duppach	0.59*	0.29*	0.47*	0.25*	0.62*	0.70*	0.31*	0.61*	0.52*	0.41*	-	0.158*	0.382*
	Longuich	0.50*	0.26*	0.25*	0.16*	0.64*	0.72*	0.34*	0.42*	0.54*	0.44*	0.29*	-	0.243*
	Orenhofen	0.88*	0.76*	0.87*	0.62*	1.00*	1.00*	0.67*	1.00*	0.030	0.72*	0.63*	0.65*	-
**d) *M. bechsteinii***														
			**LF**								**RP**			
	**Host mtF**_**ST**_**\F**_**ST**_		**ES**	**GB2**	**GS1**	**HB**	**IB3**	**RT**	**SB**		**BI**		**DU**	
LF	Blutsee		0.023	0.004	0.005	0.008	0.008	0.011	0.006		0.072		0.016	
	Einsiedeln		-	0.036*	0.032*	0.044	0.032	0.03	0.036*		0.5		0.356*	
	Guttenberg 2		0.245*	-	0.007	0.010*	0.002	0.010*	0.006		0.448		0.445*	
	Gramschatz 1		−0.001	0.252*	-	0.018*	0.005	0.004	0.004		0.488		0.351	
	Höchberg		0.075	0.142*	0.087*	-	0.009	0.028*	0.01		0.375		0.341*	
	Irtenberg 3		0.260*	0.239*	0.259*	0.148*	-	0.014	0.005		0.318		0.28	
	Reutholz		0.388*	0.373*	0.412*	0.304*	0.201*	-	0.014		0.072		0.211	
	Steinbach		0.333*	0.274*	0.320*	0.195*	0.004	0.205*	-		0.333		0.312	
RP	Bitburg		0.028	0.004*	0.009*	0.017*	0.009*	0.015	0.012*		-		0.013	
	Duppach		0.042*	0.015*	0.013*	0.030*	0.022*	0.010*	0.019*		0.311*		-	

Pairwise Φ_ST_ (below the diagonal) and F_ST_-values (above the diagonal) for a) *Spinturnix myoti*, b) *Myotis myotis* c) *Spinturnix bechsteini* d) *Myotis bechsteinii*. For *M. bechsteinii* values below the diagonal are pairwise F_ST_-values of mitochondrial microsatellites. Asterisks indicate significant differentiation (p < 0.05).

The 697 bp fragment analysed in *S. myoti* represents an overlap of 72% with the 513 bp fragment of *cytb* previously analysed for *S. bechsteini* (Figure 2b; [23]). A comparison of the most common haplotypes of each mite species showed 93% similarity between species. The overall structure of the haplotype networks does differ in that *S. bechsteini* has far fewer haplotypes (23 vs. 49) despite a larger sample size, and does not exhibit two distinct haplotype clusters. This indicates that the pattern observed in *S. myoti* may be the result of admixture from the two glacial refugia of its host.

### Mite nucDNA

No linkage disequilibrium was found between loci in either mite species. All six *S. myoti* populations showed highly significant deviations from Hardy-Weinberg equilibrium (p < 0.0001), as a result of heterozygote deficiency (F_IS_: 0.191-0.398). Similarly, *S. bechsteini* populations were not in Hardy-Weinberg equilibrium as a result of heterozygote deficiency (p < 0.0001), with five exceptions (ES-2007, GB2-2007, HB-2007, IB3-2002, OH). Microchecker indicated the possible presence of null alleles in all microsatellite loci for both species, but found no evidence for allelic dropout or stuttering. As discussed in the original marker description [36], we believe these heterozygote deficiencies are not the result of null alleles for several reasons. First, all markers show similarly heterozygote deviations indicating that null alleles would have to be present in all markers across both species. Second, mite populations may exhibit substantial inbreeding, as often observed in obligate parasite species eg. [50], as populations are often small and largely isolated. Therefore, although the possible presence of null alleles cannot be completely dismissed, we believe the markers to be biologically informative.

### *S. myoti* nucDNA

Overall genetic variation in *S. myoti* was very high with total number of alleles per locus ranging from 13.13 to 18.13, and allelic richness ranging from 12.74 to 16.75 (Table 2a). A large proportion of these alleles were specific to one bat colony, with the number of private alleles ranging from 6 in Meiringen, to 22 in Aglié (mean = 13.17, Table 2a). Between bat colonies, *S. myoti* pairwise F_ST_-values (Table 3a) showed very little differentiation (0.002 – 0.026), and significant pairwise differentiation was only found between Aglié (separated from the other colonies by the alps) and all other colonies, and between Satigny and two other colonies (Meiringen and St. Ursanne). Pairwise F’_ST_- and G”_ST_-values were slightly higher (−0.003-0.241 and 0.031-0.264 respectively) but did not differ strongly from the pairwise F_ST_-values (data not shown). An AMOVA analysis found the vast majority of variation within mite populations (98.9%; Table 4). Unlike in the mtDNA sequence, a significant, but very weak, correlation between genetic and geographic distances (β = 0.005, r^2^ = 0.362, p = 0.027; Additional file 1) was found, but *S. myoti* nuclear and mitochondrial genetic distance were nevertheless also significantly correlated (r^2^ = 0.26, p = 0.049).

**Table 4:** Analysis of Molecular variance (AMOVA) for both parasite species

**Source of variation**	**d.f.**	**Sum of squares**	**Variance components**	**Percentage of variation**
*Spinturnix myoti*				
Among colonies	5	30.96	0.04	1.1*
Among individuals, within colonies	111	512.39	0.96	26.0*
Within individuals	117	315.5	2.69	**72.9**
*Spinturnix bechsteini*				
Among regions	1	40.39	0.05	2.4*
Among colonies, within regions	11 (12)	269.92 (309.64)	0.5 (0.52)	22.5* (23.3*)
Among individuals, within colonies	289	494.33 (518.55)	0.05 (0.08)	2.2* (3.4*)
Within individuals	302	487 (496)	1.61 (1.64)	**72.9 (73.3)**

Analysis of molecular variance for a) *Spinturnix myoti* and b) *Spinturnix bechsteini*. For *S. bechsteini* a hierarchical AMOVA including region as an additional factor is shown, with the values of a non-hierarchical AMOVA given in parentheses. Asterisks indicate significant contributions.

### *S. bechsteini* nucDNA

Overall genetic variation in *S. bechsteini* was lower than that of *S. myoti* with 2.6 to 12.0 alleles per locus (mean = 6.83), and a mean allelic richness of 5.52 (Table 2c). The number of private alleles was also lower (0–10, mean = 1.79). However, it is important to note that the number of *S. bechsteini* populations was also much higher than in *S. myoti* thereby increasing the chance that we had sampled rare but widespread alleles at multiple locations.

Pairwise F_ST_-values among populations were much higher in *S. bechsteini* than in *S. myoti* (0.052-0.475, mean = 0.228; Table 3c) as well as those of its host (Table 3d), and all *S. bechsteini* populations were significantly differentiated from one another. Pairwise F’_ST_- and G”_ST_-values showed even more exacerbated differentiation between mite populations (0.333-0.929 and 0.334-0.93 respectively), but again closely followed the pattern seen in the pairwise F_ST_-values (data not shown). An AMOVA revealed very similar patterns as in *S. myoti*, with the majority of the sampled variation found within *S. bechsteini* populations (73.3%; Table 4). Nevertheless, there was also significant differentiation among mite populations (p < 0.001).

Between the spatially distant regions of Lower Franconia (LF) and Rhineland-Palatine (RP), 45% of the alleles were shared. Pairwise F_ST_-values of *S. bechsteini* between bat colonies from differing regions were very similar to those between bat colonies from the same region (within LF = 0.215, within RP = 0.235, between regions = 0.236). Indeed, a hierarchical AMOVA including regions found only 2.4% of the variation between regions, with the vast majority of variation found within *S. bechsteini* populations (72.9%) and among bat colonies within regions (22.5%; Table 4). No correlation was found between geographic and genetic distance (r^2^ = 0.027, p = 0.39; Additional file 1).

A clear temporal genetic differentiation (F_ST_ = 0.085-0.254, mean = 0.147) in *S. bechsteini* was evident in all five bat colonies sampled for mites in 2002 and 2007. This level of differentiation is comparable to the average differentiation seen between all sampling events between different bat colonies within one year (0.146 and 0.238 for 2002 and 2007 respectively). Pairwise differentiation between the five colonies was not correlated between 2002 and 2007 (Spearman’s rank correlation: r = −0.360, p = 0.238), indicating no stable sub-structuring within the region. Within individual bat colonies, only 26.0-41.2% of alleles were conserved across sampling events, also indicating strong temporal turnover of the *S. bechsteini* populations.

In all, our results for nucDNA closely mirror those of the previously analysed mtDNA [23]. It is therefore unsurprising that a significant correlation between nuclear and mitochondrial F_ST_-values can be seen (r^2^ = 0.34, p = 0.001).

### Comparison with host

**Figure 3:**
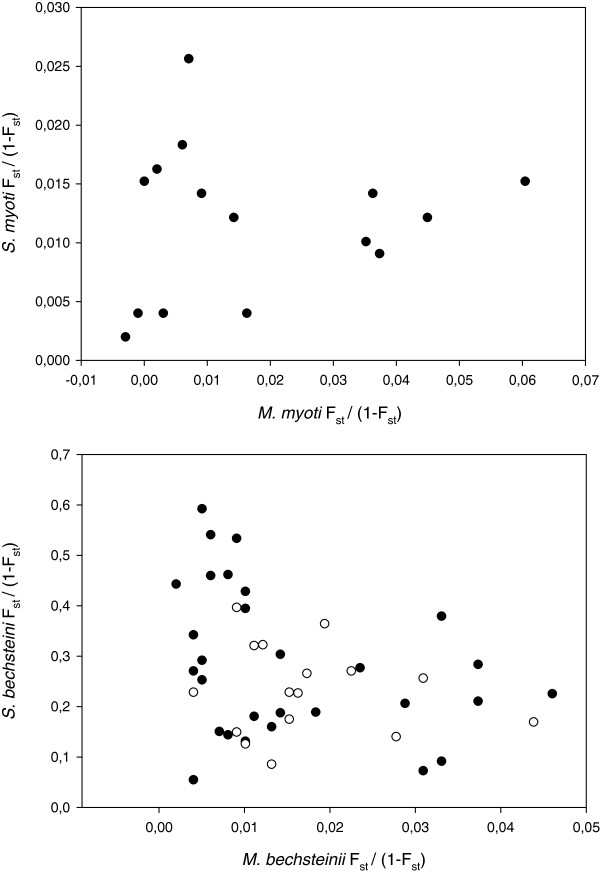
**Comparison of host and parasite genetic difference.** Pairwise genetic distance (F_ST_/1 – F_ST_) estimates between populations of hosts and parasites for **a)***Myotis myotis* and *Spinturnix myoti*, and **b)***Myotis bechsteinii* and *Spinturnix bechsteini*. For the latter pair, the symbols indicate whether colonies were within the same region (closed circles) or from different regions (open circles).

We applied mantel test analysis to examine the correlation between the genetic distances of hosts and parasites (Table 5). In neither of the host-parasite pairs were any of the pairwise Φ_ST_ or F_ST_-values significantly correlated between species after correcting for geographic distance. A plot of the pairwise genetic distance (F_ST_/1 – F_ST_) between populations of hosts and parasites reveals that this lack of correlation is due to large variation in parasite pairwise differentiation at low levels of host genetic differentiation in both species pairs (Figure 3a,b). Additionally, in *M. bechsteinii* and *S. bechsteini*, no effect of geographic distance on host and parasite pairwise genetic differentiation can be observed (Figure 3b).

**Table 5:** Correlation between host and parasite genetic distance

	***S. myoti/M. myotis***		***S. bechsteini/M. bechsteinii***	
	**β**	**R**^**2**^	**β**	**R**^**2**^
Mite mtDNA - Host mtDNA	−2.59	0.139	−0.329	0.115
Mite mtDNA - Host nDNA	−0.17	0.125	−3.33	0.025
Mite nDNA - Host mtDNA	0.003	0.022	−0.101	0.097
Mite nDNA - Host nDNA	0.343	0.013	−0.04	0.08

Correlations (partial mantel) between host and parasite genetic differentiation corrected for geographic distance. For each pair the slope (β) and the variance explained by the model (R^2^) are given. No correlations were significant after correcting for distance.

**Figure 4:**
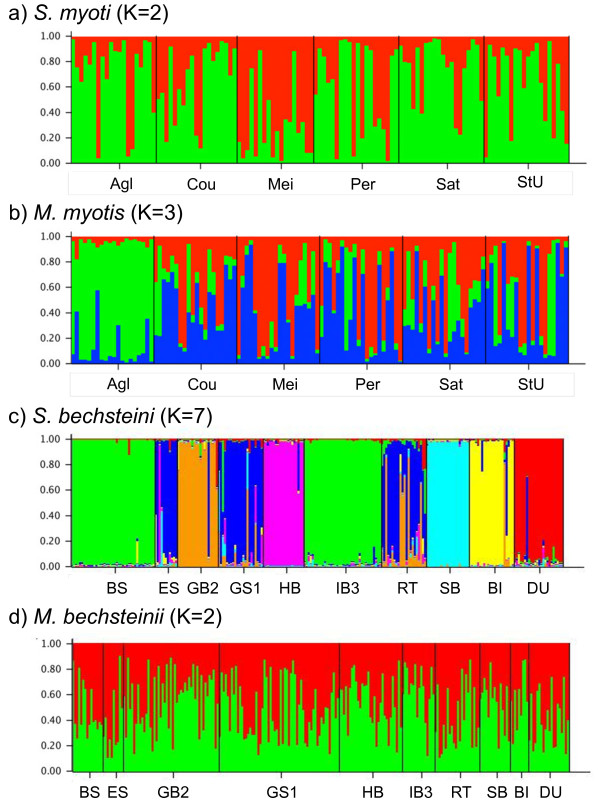
**STRUCTURE analysis for all species.** STRUCTURE results for **a)***Spinturnix myoti*, **b)***Myotis myotis***c)***Spinturnix bechsteini***d)***Myotis bechsteinii*. Colony names correspond to the abbreviations given in Tab. 2. Results are shown for the number of subpopulations with the best ΔK and highest log likelihood (*S. bechsteini* K = 7, *M. myotis* K =3). In *S. myoti* and *M. bechsteinii* no discernable population sub-structuring was found, with strongest support being found for K = 1. For these species K = 2 is shown to illustrate the lack of structuring when multiple subpopulations are assumed.

A STRUCTURE analysis revealed several interesting differences in both pairs of host and parasite (Figure 4). In *S. myoti* no clear sub-structuring was observed (Figure 4a), whereas in its host, *M. myotis*, the southernmost colony (Aglié) appears to cluster separately (Figure 4b). This is concordant with phylogeographic analyses, which have indicated that populations of *M. myotis* south of the Alps originate from a different glacial refugium [19], although this barrier is evidently not present for its mites. In *M. bechsteinii* and *S. bechsteini* clear sub-structuring according to host colony can be observed in the parasite (Figure 4c), while host colonies show no evidence for population sub-structuring (Figure 4d) as is expected with an outbreeding mating system such as swarming [16].

Finally, since host colony size between species appears to have a large effect on the population genetic structure of its parasites, we used Spearman’s rank correlation tests to investigate whether variation in host colony size within each host was correlated with parasite diversity indices (Table 6). Host colony size was not significant correlated with any of the parasite genetic indices after Bonferroni correction, although there was a positive trend between parasite allelic richness and host colony size in both species pairs.

**Table 6:** Correlation between host colony size and parasite genetic parameters

	***M. myotis***		***M. bechsteini***	
**Parasite trait**	**ρ**	***p***	**ρ**	***p***
Number of haplotypes	0.75	0.09	0.28	0.26
Nucleotide diversity	−0.26	0.61	0.29	0.24
Expected heterozygosity	0.64	0.17	0.49	0.12
Observed heterozygosity	0.41	0.43	0.16	0.64
Allelic richness	0.9	0.015	0.45	0.06

Spearman rank correlations between host colony size and parasite genetic parameters. For each pair the correlation coefficient (ρ) and the P-value are given. No correlations were significant after Bonferroni correction (p < 0.01).

## Discussion

The population genetic structure of parasite populations is strongly influenced by both its own life history as well as the life history and social system of its host. Here, we compared the population genetic structure of both host and parasite in two closely related systems where parasite life histories are nearly identical, but hosts differ substantially in social system. We find that the population genetic structure of the two mite species differs strongly as a result of the social system of their hosts, which is in agreement with our predictions based on the differences in social system of their hosts.

### Parasite population genetic structure

In *S. myoti*, overall mtDNA haplotype diversity (49 haplotypes in 120 individuals) and nuclear genetic diversity were high (mean 16.69 alleles per locus in only 20 individuals per colony). This diversity is higher than that seen in its host, *M. myotis*, and may be the result of admixture between parasites originating from several glacial refugia or species, coupled with sufficiently large host maternity colony size to prevent strong bottlenecks in the mites. Between *M. myotis* colonies, we found very low genetic differentiation and no evidence for population substructuring in the mites. Indeed the vast majority of genetic diversity of *S. myoti* occurred within bat maternity colonies suggesting a large amount of parasite exchange between hosts originating from different maternity colonies outside of the maternity period, resulting in a highly diverse and panmictic population. These results are concordant with the observation of other empirical studies on host-parasite co-variation eg. [9], that have found levels of parasite gene flow much higher than originally predicted [51]. Notably, the extensive mixing of parasites outside of the breeding period has also been found in ectoparasitic lice of birds, where the genetic structure of parasites was largely shaped by the dispersal of lice at communal wintering sites [10].

The population genetic structure of *S. bechsteini* contrasted sharply with that of *S. myoti*. Overall nuclear genetic diversity was lower, but still higher than that of its host, *M. bechsteinii*. All *S. bechsteini* populations were significantly differentiated from one another, and it was possible to assign mites to subpopulations according to their host colony of origin (Figure 4). This is likely to be due to strong genetic drift within host colonies as a result of their small maternity colony size as well as their tendency to hibernate without body contact with conspecifics. Both factors combine to drastically limit the number mites present within a host colony when the colonies reform in spring. Nevertheless, *S. bechsteini* populations within colonies were temporally unstable (between sampling years mean F_ST_ = 0.147 for nucDNA) suggesting that gene flow between colonies is still substantial. In agreement with this finding, Bruyndonckx *et al.*[23] observed a large turnover in haplotypes between sampling years. Despite this strong differentiation among colonies and years at a local scale, differentiation of *S. bechsteini* between colonies from spatially distinct regions was not higher than within regions, suggesting that overall genetic diversity of the *S. bechsteini* population at a regional level is Stable. A comparable pattern of strong population genetic substructure and high levels of genetic drift is also seen in the parasites of other host taxa with similarly closed societies as in Bechstein’s bats, e.g. in the ectoparasitic chewing lice of pocket gophers rev. in [11].

### Relationship between parasite and host population structure

No correlation was found between host and parasite genetic differentiation in either of the species. Additionally, within each of the two host species, colony size did not significantly correlate with any of the parasite genetic indices, although a trend of increasing allelic richness with increased colony size was present in both species pairs.

### Influence of host social system

Our results indicate that differences between hosts in colony size, mating system, and in particular the degree of social interaction outside of the summer maternity roosts have a large effect on parasite genetic structure. *M. bechsteinii* has a remarkably closed social system, with very strong natal philopatry and vastly limited social interaction outside of the summer maternity season [52,53]. Previously, it has also been shown that *M. bechsteinii* actively reduces the intensity of another ectoparasite species that deposits its larval stages in the roosts via roost-switching behaviour (bat flies; [54]). It thus seems possible that the much more closed social system of *M. bechsteinii,* as compared to *M. myotis,* has also evolved to restrict the infestation of other parasites, such as wing mites, that depend on body contact between hosts.

In *M. myotis*, our data provide no evidence for such anti-parasite behaviour. While this may be because there is an insufficient number of alternative roosts to increase population subdivision into smaller colonies, social interaction during other periods such as mating and hibernation is also extensive, thereby further permitting parasite exchange.

Several other factors probably have also influenced the genetic structure of both mite species, but these factors do not detract from the influence of host social structure. For example, the phylogeographic history of both mite species is quite different, and may explain the differences in the number of haplotypes and nucleotide diversity found in the mtDNA sequences (compare Figure 2a and Figure 2b), and likely also the diversity found in the nucDNA microsatellites. Nevertheless, these macrogeographic differences between the two mite species cannot explain the differences seen in population genetic structure on a microgeographic scale. For example, almost all *M. bechsteinii* colonies harboured *S. bechsteini* populations that were monotypic or had only two haplotypes as a result of the strong winter bottleneck. This contrasts sharply with the sampled populations of *S. myoti*, in which we found between seven and fourteen haplotypes in only twenty samples per populations. Thus, although the history of both species has certainly influenced the overall nucleotide and genetic diversity observed, the differences in genetic structure observed on a population level are still primarily the result of differences in host social system. Another major difference between the two mite species is the potential use of secondary host, with identical social system, in *S. myoti*. Here too, the social systems of the hosts play a role, as the closed social system of *M. bechsteinii* minimizes contact between conspecifics and virtually eliminates contact with other species. Therefore, while the possible use of additional hosts in *S. myoti* may increase its effective population size and potentially also increase its dispersal opportunities, these factors can also be (indirectly) attributed to the differing social systems of the hosts.

### Consequences for parasite evolutionary potential

The observed differences in population genetic structure of the two mite species should strongly influence their evolutionary potential. In *S. bechsteini*, the influence of genetic drift strongly limits the likelihood that any local adaptation to its host maternity colony is able to persist and spread. *S. myoti*, in contrast, has a much more stable population genetic structure as well as a higher rate of gene flow relative to its host, and may therefore be able to locally adapt to its major host, *M. myotis*.

The observed difference in population genetic structure between the mites analysed here also have broader consequences for general investigations of parasites in relation to host social system. For example, they suggest that parasite species with very similar life histories may have vastly different evolutionary potentials depending on not only the life history but also the social system of their hosts. As a result, we conclude that host social system can be effective beyond reducing the overall chance of parasite infection, by potentially also playing a role in limiting the evolutionary potential of, often unavoidable, parasites. Therefore, comparative and theoretical studies of host-parasite interactions that investigate the role of host population size and community modularity eg [55] will be critical not only in understanding parasite transmission, but also in investigating the evolutionary potential of established parasite species.

## Conclusions

In conclusion, our results suggest that host social system can strongly influence parasite population genetic structure. Most notably, host maternity colony size appears to strongly affect the genetic drift experienced by the different parasite species and the social organization of the host outside of the maternity period affects the opportunities for parasite exchange between individuals from remote maternity colonies. We conclude that such differences in host social system have consequences for both the direct costs of parasites as well as the general threat of disease transmission. Therefore, the concurrent genetic analysis of host and parasite allows for inferences about the movements and social contacts of host species, as well as broader conclusions regarding host-parasite dynamics.

## Availability of supporting data

The datasets for *S. myoti* and *S. bechsteini* supporting the results of this article may be requested from the corresponding author. The haplotype sequences for *S. myoti* have been deposited in GenBank under ascension numbers [KJ174107-KJ174155].

## Competing interests

The authors declare that they have no competing interests.

## Authors’ contributions

All authors designed the research and participated in sample collection; JvS and NB performed the molecular genetic studies and data analysis; and JvS supported by all authors wrote the paper. All authors read and approved the final manuscript.

## Supplementary Material

Additional file 1 Supplementary analyses to: The effect of host social system on parasite population genetic structure: comparative population genetics of two ectoparasitic mites and their bat hosts. Click here for file
