# Supplementary material for: The effect of host social system on parasite population genetic structure: comparative population genetics of two ectoparasitic mites and their bat hosts
Source: BMC Evol Biol. 2014 Jan 30;14:18. doi: 10.1186/1471-2148-14-18 (PMC3925363; doi:10.1186/1471-2148-14-18)

Additional File 1

(Belongs to: van Schaik J, Kerth G, Bruyndonckx N, Christe P: **The effect of host social system on parasite population genetic structure: comparative population genetics of two ectoparasitic mites and their bat hosts.** submitted to BMC Evolutionary Biology)

Previously analysed samples:

Below is an overview of which markers were used in this study and where a complete description of their amplification conditions can be found.

Species	Markers used	Primers/markers described in	Data originally published in
S. myoti			
mtDNA	cytB (697bp)	Simons *et al.*, 1994	this study
nucDNA	SM7, SM11, SM13, SM17, SM18, SM19, SM51, SM55	van Schaik *et al.*, 2011	this study
M. myotis			
mtDNA	HV-2 (307bp)	Castella *et al.*, 2001	Castella *et al.*, 2001*
nucDNA	A13, B11, B22, C113, E24, F19, G9, H19, H29, G30	Castella & Ruedi 2000	Castella *et al.*, 2001*
S. bechsteini			
mtDNA	cytB (513bp)	Bruyndonckx *et al.*, 2009	Bruyndonckx *et al.*, 2009
nucDNA	SM11, SM16, SM17, SM18, SM35	van Schaik *et al.*, 2011	this study
M. bechsteinii			
mtDNA	mtMicrosatellites (AT1, AT2)	Kerth *et al.*, 2000	Kerth *et al.*, 2000
nucDNA	B15, B22, B23, G30, P5, P8, P20, paur3	Kerth *et al.*, 2002	Kerth *et al.*, 2003

* = plus two new colonies in this study

Structure

The program Structure was used to evaluate population sub-structuring for each species. Below are the Log-likelihood values for all tested K’s for each species, as well as the concordant DeltaK values (as described in Evanno et al., 2005) used to determine the most likely number of sub-populations.

Likelihood of all K values run for all species (calculated using StructureHarvester):

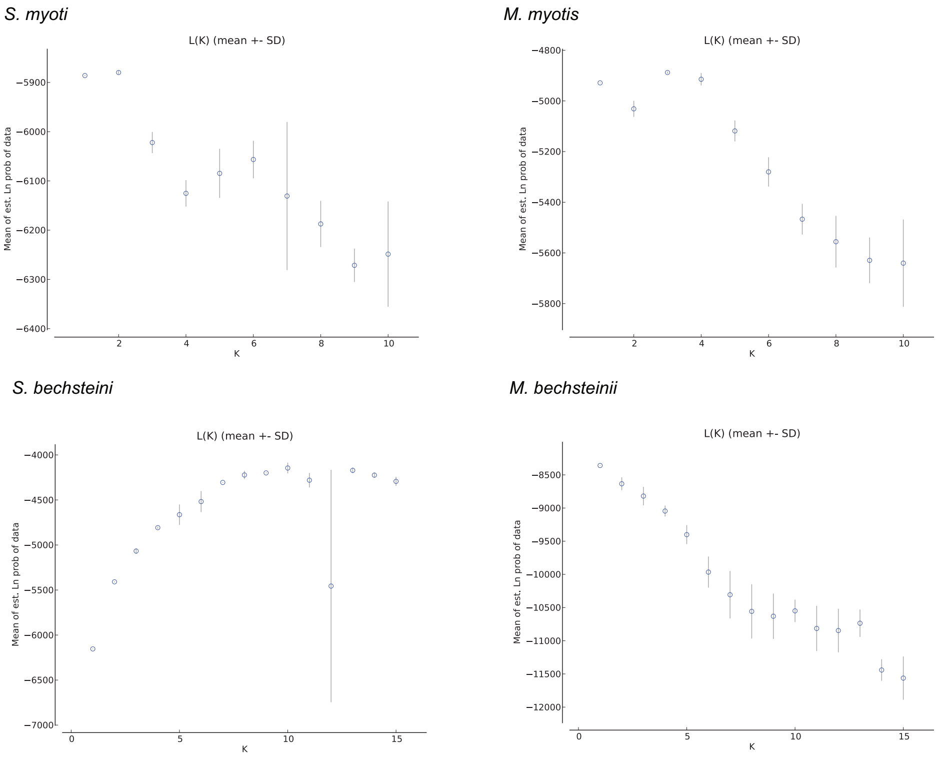

DeltaK values for all species (calculated using StructureHarvester):

S. myoti

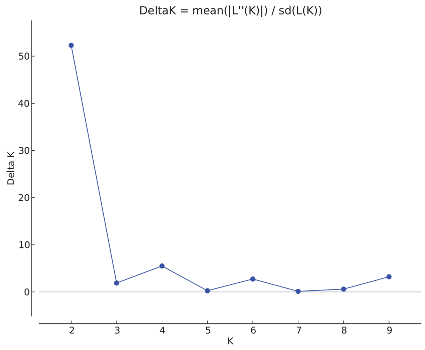

M. myotis

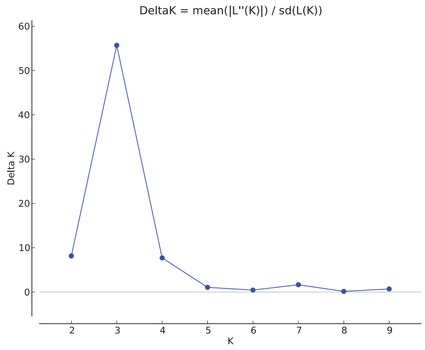

S. bechsteini

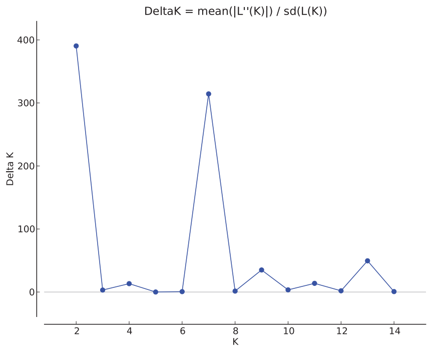

M. bechsteinii

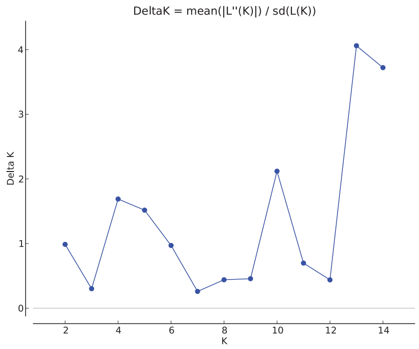

As can be seen in the graphs for *S. bechsteini* both K=2 and K=7 resulted in high DeltaK values, and K=10 (the actual number of populations sampled) resulted in the highest overall Log-likelihood. Below, outputs for K=2 and K=10 are provided (K=7 can be found in Figure 4).

S. bechsteini (K=2)

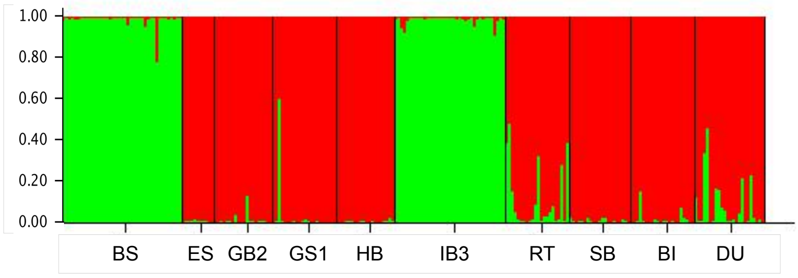

S. bechsteini (K=10)

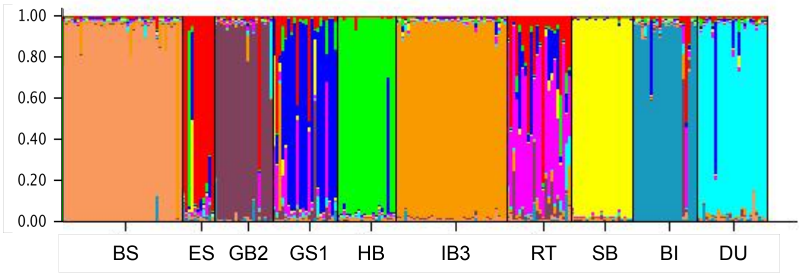

Isolation by distance

Below are the statistical correlations and graphs correlating nuclear genetic distance and geographic distance (using F_{ST} / (1- F_{ST}) and Ln(distance)). For both *S. myoti* and *M. myotis* genetic distance is significantly (positively) correlated with geographic distance. No such relationship is found in *S. bechsteini* or *M. bechsteinii*, nor is it found when only samples within LF are analysed.

	β	R^2
S. myoti	0.005	0.362*
M. myotis	0.015	0.342*
S. bechsteini	-0.012	0.027
M. bechsteinii	0.001	0.026

S. myoti

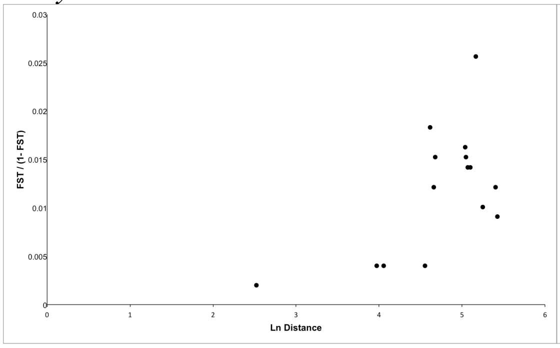

M. myotis

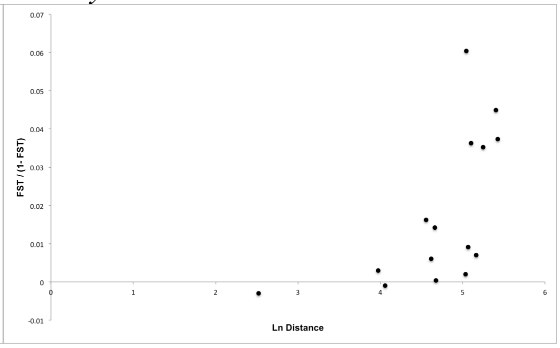

S. bechsteini

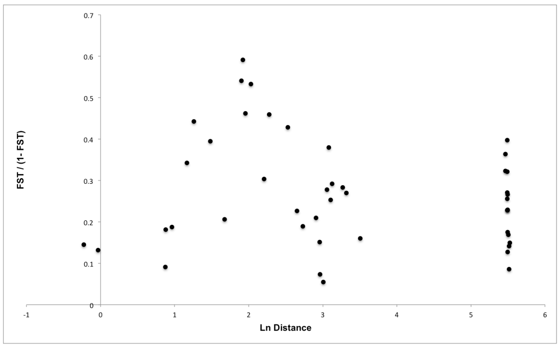

M. bechsteinii

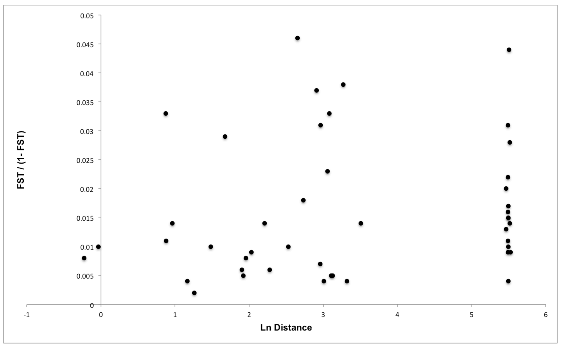

S. bechsteini (without RP)

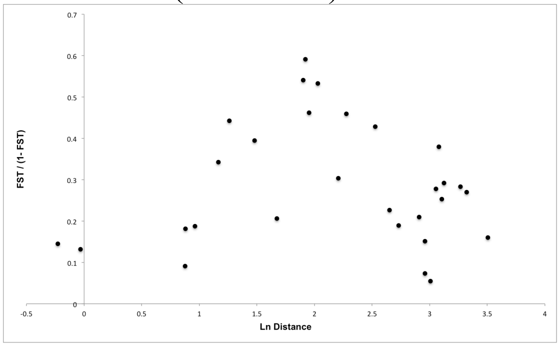

M. bechsteinii (without RP)

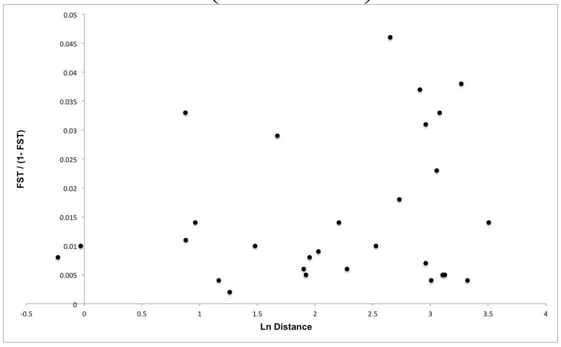

Supplement: Additional file 1 — Supplementary analyses to: The effect of host social system on parasite population genetic structure: comparative population genetics of two ectoparasitic mites and their bat hosts. [file 1471-2148-14-18-S1.pdf]
